# Non-Alcoholic Fatty Liver Disease: Implementing Complete Automated Diagnosis and Staging. A Systematic Review

**DOI:** 10.3390/diagnostics11061078

**Published:** 2021-06-12

**Authors:** Stefan L. Popa, Abdulrahman Ismaiel, Pop Cristina, Mogosan Cristina, Giuseppe Chiarioni, Liliana David, Dan L. Dumitrascu

**Affiliations:** 1 2nd Medical Department, “Iuliu Hatieganu” University of Medicine and Pharmacy, 400006 Cluj-Napoca, Romania; popa.stefan@umfcluj.ro (S.L.P.); lilidavid2007@yahoo.com (L.D.); ddumitrascu@umfcluj.ro (D.L.D.); 2 Department of Pharmacology, Physiology and Pathophysiology, Faculty of Pharmacy, “Iuliu Hațieganu” University of Medicine and Pharmacy, 400349 Cluj-Napoca, Romania; pop.cristina@umfcluj.ro (P.C.); cmogosan@umfcluj.ro (M.C.); 3 Division of Gastroenterology, University of Verona, 1-37126 AOUI Verona, Italy; chiarioni@alice.it

**Keywords:** non-alcoholic fatty liver disease (NAFLD), metabolic associated fatty liver disease (MAFLD), non-alcoholic steatohepatitis (NASH), artificial intelligence (AI), machine learning, automated diagnosis

## Abstract

Background: Non-alcoholic fatty liver disease (NAFLD) is a fast-growing pathology around the world, being considered the most common chronic liver disease. It is diagnosed based on the presence of steatosis in more than 5% of hepatocytes without significant alcohol consumption. This review aims to provide a comprehensive overview of current studies of artificial intelligence (AI) applications that may help physicians in implementing a complete automated NAFLD diagnosis and staging. Methods: PubMed, EMBASE, Cochrane Library, and WILEY databases were screened for relevant publications in relation to AI applications in NAFLD. The search terms included: (non-alcoholic fatty liver disease OR NAFLD) AND (artificial intelligence OR machine learning OR neural networks OR deep learning OR automated diagnosis OR computer-aided diagnosis OR digital pathology OR automated ultrasound OR automated computer tomography OR automated magnetic imaging OR electronic health records). Results: Our search identified 37 articles about automated NAFLD diagnosis, out of which 15 articles analyzed imagistic techniques, 15 articles analyzed digital pathology, and 7 articles analyzed electronic health records (EHC). All studies included in this review show an accurate capacity of automated diagnosis and staging in NAFLD using AI-based software. Conclusions: We found significant evidence demonstrating that implementing a complete automated system for NAFLD diagnosis, staging, and risk stratification is currently possible, considering the accuracy, sensibility, and specificity of available AI-based tools.

## 1. Introduction

Non-alcoholic fatty liver disease (NAFLD) is a fast-growing pathology worldwide, being considered the most common chronic liver disease. It is diagnosed based on the presence of steatosis in more than 5% of hepatocytes without significant alcohol consumption. The term NAFLD consists of the spectrum ranging from the benign form known as non-alcoholic fatty liver (NAFL) and, possibly, deteriorating to the harsher form called non-alcoholic steatohepatitis (NASH) [1,2,3].

Even though NAFLD is present also in normal weight patients, more than 80% of NAFLD patients are obese, having a body mass index (BMI) > 30 kg/m^2^ [3,4,5,6]. NAFLD prevalence has been recently expanding globally, along with several metabolic diseases including obesity. NAFLD is expected to become the most frequent cause for liver transplant in less than a decade. Its prevalence ranges from 6%–35% of the global population, and approximately 30% of the United States are diagnosed with NAFLD (90 million persons) [3,4,5,6].

Diagnosing NAFLD is laborious because the currently feasible serological and imagistic methods are not able to make a distinction between steatosis and NASH [3,4,5,6]. Liver biopsy is a technique capable of a precise diagnosis. Unfortunately, it is rarely recommended in clinical practice because of the increased risk of severe bleeding and life-threatening complications [3,4,5,6]. An alternative to liver biopsy are the recently developed imagistic methods, with a high diagnosis accuracy: magnetic resonance imaging (MRI) with proton density fat fraction (PDFF) and proton magnetic resonance spectroscopy (1 H-MRS) [2,3,4].

The main domains in healthcare in which artificial intelligence (AI) applications are being used include diagnostic imaging, laboratory data, electrodiagnosis, electronic health records, and records from wearable devices. For this reason, our systematic review aims to provide a comprehensive overview of important studies of AI applications, which might help physicians in the management of NAFLD. Furthermore, we compared human expert NAFLD diagnosis accuracy, specificity, and sensitivity with automatic diagnosis systems, in case of the available selected studies provided this particular information.

## 2. Materials and Methods

**Figure 1:**
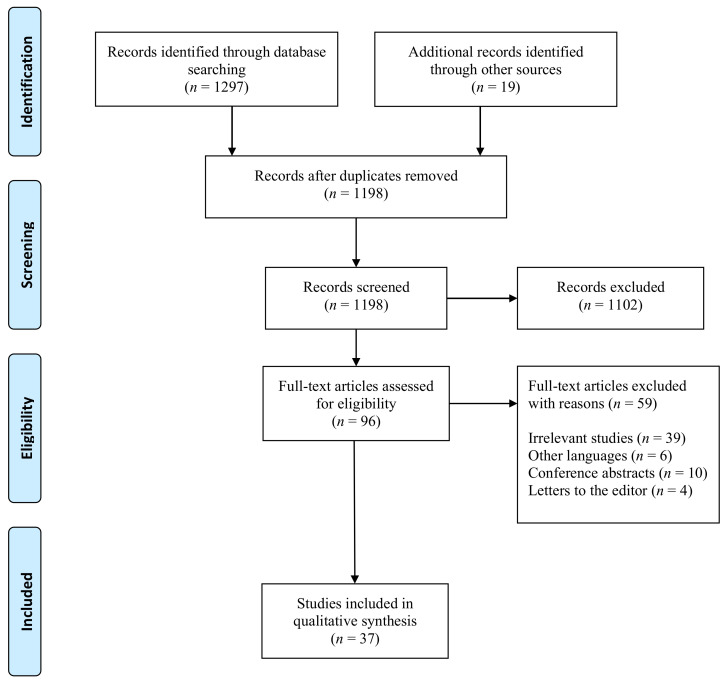
Flow diagram for study selection.

PubMed, EMBASE, Cochrane Library, and WILEY databases were filtered for relevant publications regarding AI applications in NAFLD. The search terms included: (non-alcoholic fatty liver disease OR NAFLD OR NAFL) AND (artificial intelligence OR machine learning OR neural networks OR deep learning OR automated diagnosis OR computer-aided diagnosis OR digital pathology OR automated ultrasound OR automated computer tomography OR automated magnetic imaging OR electronic health records). Exclusion criteria were: case reports, abstracts, letters to the editor, conference presentations, pediatric studies, studies written in languages other than English, and editorials (Figure 1).

Two independent authors (S.L.P and A.I.) reviewed for eligibility titles, abstracts, and full text of eligible articles. Data extraction was also conducted independently by both reviewers. Extracted data on the “authors” names, year of publication, country or study population, sample size, study design, gender ratio, number and percentage of NAFLD patients, the method used to diagnose NAFLD, and artificial intelligence-based method were reported into three separate tables. Figure 1 shows the search strategy using the PRISMA flow diagram.

## 3. Results

The initial search retrieved a total of 1297 studies. We screened a total of 96 studies, and we excluded 59 articles as follows: irrelevant original studies to this review topic (*n* = 46), other languages (*n* = 6), conference abstracts (*n* = 4), and letters to the editor (*n* = 3). Finally, 37 articles fulfilled our inclusion and exclusion criteria and were included in the systematic review as demonstrated in Figure 1. This section may be divided by subheadings. It should provide a concise and precise description of the experimental results, their interpretation, as well as the experimental conclusions that can be drawn.

### 3.1. Artificial Intelligence Based Imaging for NAFLD Diagnosis

The complex process of developing machine-learning software for automated radiological diagnosis entails image reconstruction, segmentation, detection, data mining, and quantification and, finally the clinical interpretation of raw digital information about on shape, texture, volume, diffusion, and other parameters [7,8,9,10,11]. The main factors which are accelerating the transition from traditional radiology to AI-based radiology worldwide include reduced diagnosis errors, instant diagnosis with no delays, a higher accuracy compared to human radiologists, and, probably the most important factor, a considerably lower cost [7,8,9,10,11,12,13].

We found 15 articles analyzing automated diagnosis using imaging techniques in NAFLD (Table 1 and Table 2). In order to compare 3 automated diagnosis techniques using ultrasound image, Cao et al. included in their study 240 subjects organized into 4 groups: healthy controls, mild NAFLD, moderate NAFLD, and severe NAFLD [14]. The ultrasound images were evaluated using several methods including envelope signal, grayscale signal, and deep-learning index. The following parameters were compared between the 4 groups: draw receiver operating characteristic (ROC) curves and the area under the curve (AUC) [15]. The study reported that all the methods used in the study were associated with a significant ability for an automated diagnosis in NAFLD (AUC > 0.7) and that deep-learning index exhibited the best diagnostic capability to differentiate between moderate and severe NAFLD (AUC = 0.958) and demonstrated best sensitivity as well as specificity for NAFLD staging [15]. The authors concluded that deep learning software is able to recognize and stage NAFLD using two-dimensional hepatic ultrasound images, demonstrating the feasibility of a complete automated imagistic diagnosis [15].

Because the imagistic differential diagnosis between fatty liver disease and cirrhosis using ultrasound may be troublesome, Acharya et al. analyzed the possibility of automated software-based on curvelet transform method to discriminate between the normal liver, fatty liver disease, and cirrhosis [16]. The study protocol included the following features to be extracted from curvelet transform coefficients: higher-order spectra (HOS) bispectrum, HOS phase, fuzzy, Kapoor, max, Renyi, Shannon, Vajda, and Yager entropies [16]. Results demonstrated that the automated system could diagnose normal liver and fatty liver disease, in addition to cirrhosis through the utilization of a probabilistic neural network classifier, reporting an accuracy, sensitivity, and specificity of 97.33%, 96%, and 100%, respectively [16]. Those successful results were obtained using only six parameters by the automated diagnosis neural network [16]. The study clearly demonstrates that a precise diagnosis for chronic liver disorders is actually available for current clinical practice and also for experimental studies.

Biswas et al. evaluated the efficiency of a deep learning (DL) software with a power of seven million weights per on hepatic image obtained with ultrasound, followed by a 22 layered neural network processing [17]. The main operations performed by the Symtosis System include convolution, pooling, rectified linear unit, dropout, and inception model. The additional speed and accuracy are given by the optimization of tissue localization and the precise process of removing background information [17]. The authors also compared their results with the most frequently used conventional machine learning protocols: ELM and SVM. In the study, liver ultrasound images were obtained from 36 patients with fatty liver disease and 27 healthy controls [17].

The study reported that diagnosis and risk stratification accuracies of 82% for SVM, 92% for ELM, and 100% for DL systems, and a corresponding AUC of 0.79, 0.92, and 1.0, respectively [17]. The superiority of the DL Symtosis System was further demonstrated with the validation of two class biometric facial data showing a 99% accuracy [17]. The authors concluded that the DL Symtosis System is an accurate tool for diagnosis and risk stratification of fatty liver disease and can be used in clinical practice [17].

The Bayes factor is a summary of provided evidence favoring the likelihood ratio of one particular hypothesis to the likelihood of another through a statistical model [18]. Using objective liver ultrasound parameters which are computed in a Bayes factor, Ribeiro et al. analyzed the efficiency of a computer-aided diagnosis (CAD) for steatosis staging [18]. The study demonstrated that the Bayes automated classifier had an accuracy, sensitivity, and specificity of 93.33%, 94.59%, and 92.11%, respectively [18]. This study demonstrated that CAD system can be considered as an accurate automated system for steatosis classification without a function for diagnosis [18].

If several studies have analyzed different AI-based software as a diagnosis tool for NAFLD using ultrasound images, Kuppili et al. analyzed the possibility of risk stratification in fatty liver disease using extreme learning machine (ELM), a class of Symtosis [19]. The initiative came as a reaction to the machine learning software based on support vector machines (SVM) characterized by slow data processing and mismatch between grayscale features, followed by diagnosis and classification errors [19]. There were 63 patients included in the study, 36 patients with fatty liver disease and 27 healthy controls, and ELM was used in the process of software training based on a single-layer feed-forward neural network [19]. The study reported a significant processing speed upgrade of 40% for ELM compared with SVM. Furthermore, ELM demonstrated an accuracy of 96.75% in comparison with SVM showing an accuracy of 89.01%, with AUC: 0.97 and 0.91, respectively [19]. The authors validated their automated software for risk stratification using two-class biometric facial public data. The result of the final validation was an accuracy of 100%, the highest accuracy found from all the studies we have evaluated [19].

In a prospective study on 228 patients with chronic liver disorders, Nagy et al. evaluated the diagnosis values of a set of hepatic ultrasound characteristics, which were further analyzed with the support of an SVM classifier for steatosis [20]. The results were compared with the results obtained from the hepatic biopsy, which is still the “gold standard” technique for fatty liver disease diagnosis. Three ultrasound parameters were used by the SVM classifier: coefficient of luminance variation, median luminance, and hepato-splenic attenuation index [20]. Results demonstrated good accuracy of 89.17% for the method, with an AUC of 0.923 for division of liver steatosis in two stages (mild vs. moderate-severe) [20].

A study by Subramanya et al. analyzed the efficiency of CAD system for diagnosing and staging fatty liver disease on 53 ultrasound images. The authors reported a superior performance, publicizing the values of 84.9 ± 3.2 for mean accuracy and standard deviation, concluding that CAD system is a convenient tool for diagnosis and staging of fatty liver disease [21].

In a prospective study on 120 subjects with steatosis or normal liver, Mihăilescu et al. evaluated the possibility of automated steatosis staging using ultrasound images analyzed by random forests and SVM classifiers [14]. Ground truth data was represented by the diagnosis based on human experts’ ratings, and the staging of the steatosis was based on a large set of labeled images. The authors reported that random forests had better accuracy than SVM classifier [14]. A study by Han et al. evaluated a DL system based on radiofrequency data obtained from magnetic resonance imaging (MRI)/derived proton density fat fraction [14].

The DL algorithms utilized one-dimensional convolutional neural networks, applying the test group in order to to analyze the classifier for accuracy, sensitivity, specificity, positive predictive value (PPV), and negative predictive value (NPV) [22]. An accuracy of NAFLD diagnosis of 96% (95% CI: 90%, 99%), 98 of 102; sensitivity of 97% (95% CI: 90%, 100%), 68 of 70; specificity, 94% (95% CI: 79%, 99%), 30 of 32; PPV, 97% (95% CI: 90%, 99%), 68 of 70; NPV, 94% (95% CI: 79%, 98%) was reported, demonstrating that DL algorithms using radiofrequency ultrasound data have a significant accuracy for NAFLD diagnosis [22]. If the majority of the available studies are analyzing the possibility of an automated diagnosis of NAFLD using ultrasound images, only a limited number of studies [3] are evaluating the possibility of computer tomography (CT) NAFLD-based diagnosis and staging.

A retrospective study by Graffy et al. analyzed an automated volume-based liver attenuation method that was tested using three-dimensional convolutional neural networks [23]. In addition, CT fat fraction was also performed and was compared with human radiology “experts” evaluations [23]. The study included 11,669 CT scans in 9552. The authors reported that algorithm errors occurred only in 7 cases [23]. A significant number of agreements among manual and computerized measurements were found, reporting a mean difference of 2.7 HU with a median of 3 HU, and a value r^2^ = 0.9230 The study demonstrated that complete automation of NAFLD diagnosis based on CT images is possible, and the results of the automated software match significantly with the results obtained by human radiologists [23].

Jirapatnakul et al. analyzed the accuracy of an automated method performed using non-contrast low-dose chest CT (LDCT) for measuring liver attenuation and compared it with human radiologists diagnosis [24]. The protocol of the study provided an algorithm, identifying an area inferior to the right lung within the hepatic parenchyma and utilizes a sampling strategy, allowing the exclusion of non-liver parenchyma [24]. Automated measurements of liver attenuation from LDCT scans were demonstrated to be a precise automated method of diagnosis, and it can be implemented in clinical practice [24]. Further, a study by Huo et al. concluded that hepatic steatosis measured using an automatic CT-based on automatic liver attenuation ROI-based measurement (ALARM) achieved a significant match with the human radiologists’ estimation for liver steatosis [25].

Unfortunately, few studies analyzed the possibility of automated NAFLD diagnosis using imaging techniques applied to animal models. A study on rodents by De Rudder et al. showed that an automated method of measurement of steatosis based on quantification of macrovesicular steatosis area presented a significant correlation with micro-CT liver density, hepatic fat content (*r* = 0.89), steatosis scores (*r* = 0.89), and the CD36 gene expression (*r* = 0.87) [26]. The automated tool also precisely identified and quantified macrovesicular steatosis, mixed inflammation, and pericellular fibrosis [26]. The authors concluded that their automated method is a precise tool for monitoring fatty liver disease evolution. Further, a study conducted by Starke et al. analyzed the automated evaluation of hepatic ultrasound images for diagnosing fatty liver in cows [27].

The results demonstrated that computer-aided ultrasound diagnosis is able to perform an automated diagnosis of fatty liver disease in herd health programs, and the digital tool can be extended to other types of animals [27].

Acorda et al. performed a study on 158 Holstein-Friesian cows and made a comparative evaluation of hepatic fatty infiltration using liver blood tests, ultrasound images, and automated digital [28]. The authors reported that automated digital analysis had the highest sensitivity, specificity, and accuracy for diagnosing fatty liver disease [28].

### 3.2. NAFLD Diagnosis and Staging Using Digital Pathology

Digital pathology is defined as the process of digitizing histopathology slides, while utilizing whole-slide scanners, enabling acquisition, automated diagnosis, risk, and prognostic interpretation of pathology information generated from a digitized glass slide [29,30,31,32,33]. Artificial intelligence software using a considerable digital database, followed by machine learning networks, is capable of a complete automated process of diagnosis in a wide range of pathology [29,30,31,32,33].

We found 15 articles analyzing automated diagnosis using digital pathology in NAFLD (Table 3 and Table 4). We mention that we did not include in this review articles analyzing digital pathology techniques used in NASH and NAFL.

A wide range of digital algorithms and software were introduced in the last decade aiming for a complete automated diagnosis of steatosis using liver biopsy samples. Different methods have been utilized on order to automatically discriminate between steatosis and non-steatotic tissue as healthy liver tissue, hepatic tumors, blood vessels, and bile ducts.

A study by Forlano et al. was conducted on 246 consecutive patients, all with biopsy-proven NAFLD, which were followed up by used machine learning from 2010 to 2016, in order to create a complete automated software for diagnosing and quantifying steatosis, inflammation, ballooning, and fibrosis from hepatic biopsy samples [34]. Machine learning software was found to identify specific histologic NAFLD characteristics with interobserver and intraobserver agreement levels that ranged between 0.95 to 0.99 and in a subgroup of paired liver biopsy samples, the automated result generated by the machine learning software was more precise than the non-alcoholic steatohepatitis Clinical Research Network scoring system [34]. The authors of the study concluded that this software could objectively analyze anatomopathological features of NAFLD from liver biopsy samples in an extremely short period of time. These software results were found to correspond with results from histopathologists, opening the opportunity for digital pathology real-time diagnosis and a good balance between cost and efficiency [34].

Digital image analysis (DIA) is a new method for precise histological diagnosis of NAFLD, because no inter-observer variability occurs, and it has high reproducibility [35]. For this reason, Munsterman et al. developed a new DIA algorithm implemented as a Java plug-in in FIJI and can automatically process quantification of steatosis on whole-slide images (WSIs) from the liver biopsy samples using the Pathomation extension [35]. The software algorithm finds a steatosis proportionate area, and logistic regression was introduced in order to discriminate steatosis from healthy liver tissue [35]. In the study, 61 NAFLD patients and 18 healthy controls were included, and the liver biopsies were analyzed by an expert pathologist [35].

The software was programmed to find potential steatotic hepatocytes and to differentiate them from bile ducts, blood vessel and tissue tearing, using criteria of size, roundness, and color [35]. Steatotic hepatocytes were found using the following criteria: white color, specific size range, and round shape [35]. Results demonstrated an accuracy of 91.9% for the new software, and the AUC of properly identified steatosis was 0.970 (95% CI 0.968–0.973), *p* < 0.001 [35]. The authors concluded that the novel digital analysis could be used for quantification of steatosis in clinical practice but also in clinical trial studies of NAFLD [35].

A significant step forward was made by a new approach of NAFLD diagnosis using multiphoton imaging, digital three-dimensional reconstructions, and computational simulations, concerning the creation of a spatially-resolved geometrical model describing the structure of the liver, with the purpose of precise analysis of evolutive stages of fatty liver disease [36]. The study protocol provided new perspectives for the identification of multi-parametric cellular and tissue signatures in NAFLD pathophysiology and digital pathology. Several techniques were used for high-resolution reconstruction of sinusoidal networks, nuclei, lipid droplets, and hepatocytes from fluorescent image stacks of fixated liver tissue, tinted using fluorescent molecules of small size and specific antibodies [36]. In order to analyze nuclear texture, four essential features were measured: contrast, entropy, homogeneity (angular second moment), and local homogeneity (inverse difference moment) [36].

3D spatially-resolved quantitative analysis of liver biopsy was reported to uncover new morphological features in NAFLD: specific in nuclear texture in pericentral hepatocytes; quantitative changes of lipid droplets size distribution; alterations of hepatocytes apical plasma membrane; pericentral hepatocytes alteration of dipeptidyl-peptidase-4 [36].

Further, biliary fluid dynamic simulations made a statistically significant prediction of micro-cholestasis in parallel with elevated cholestasis blood tests [36]. The results of the study contribute to the expansion of high-definition medicine by introducing new concepts and histologic criteria in the process of automated NAFLD diagnosis [36].

A study conducted by Vanderbeck et al. evaluated liver biopsy samples from 59 NAFLD patients [37]. Furthermore, automatic quantification of lobular inflammation and ballooning was introduced in new software that analyzed digital images of slides involving liver biopsy samples stained using hematoxylin and eosin [37]. The study demonstrated that the automated classifier detected and quantified macrosteatosis with a precision greater than or equal to 95%, demonstrating that the implementation of automated digital pathology diagnosis for NAFLD is feasible [37]. Teramoto et al. tested an automated system for the diagnosis and classification of NAFLD liver biopsy samples based on Matteoni classification [38].

The system was depended on topological data analysis methodology in combination with linear machine learning techniques [38]. In the study were included 79 patients with NAFLD, and digital images of liver tissue samples, tinted with hematoxylin and eosin, were used to train the automated system [38]. An accuracy rate of > 90% for differentiating between the two NAFLD groups was obtained (NASH and non-NASH, respectively), and the greatest AUC from ROC analysis reached 0.946 for the purpose of identifying NASH and NAFL2 (Matteoni type 2), utilizing both 0-dimensional and 1-dimensional persistence images [38].

A study conducted by Gawrieh et al. aimed to develop automated software with the purpose of detection and quantification of hepatic fibrosis from liver biopsy samples obtained from NAFLD patients [39].

A total of 987 observations of fibrosis characteristics were obtained by two expert pathologists and were further used to train and test the machine learning models to detect fibrosis [39]. The study reported a significant correlation between the artificial intelligence-based system and the human pathologist score of fibrosis stage: the models’ calculated areas under the ROC were >90% for the purpose of detection of normal fibrosis and bridging fibrosis; 86.4% for portal fibrosis; 83.3% for pericellular fibrosis; 78.6% for periportal fibrosis [39].

The authors concluded that the excellent accuracy of the artificial intelligence-based system permits an integrated automated tool for the quantification of hepatic fibrosis in clinical practice and also in clinical trials [39].

Vanderbeck et al. conducted a study on 27 NAFLD patients and 20 healthy controls using supervised machine learning classifiers with the purpose of automatizing the classification of steatosis in NAFLD patients and other hepatic areas, which are colored in white in images of hematoxylin and eosin-stained biopsy [40]. A percentage of 89% overall accuracy of the classification algorithm was demonstrated [40].

Furthermore, macrosteatosis, bile ducts, portal veins, and sinusoids were precisely detected in >82% [40]. The high accuracy of automated identification and staging of steatosis obtained with this algorithm brings additional evidence for the feasibility of a complete automated diagnosis of NAFLD [40].

In the last two decades, several studies using experimental animal models analyzed novel techniques of implementing an automated computerized system for the diagnosis and staging of NAFLD. Using a deep learning algorithm Ramot et al. analyzed glass slides of mice liver with NAFLD, comparing the manual semiquantitative microscope-based assessment with data output obtained from the artificial intelligence software [41].

The algorithm diagnosed precisely the fatty vacuoles percentage, showing an important association (*r* = 0.87, *p* < 0.01) among the semiquantitative techniques performed by a human pathologist and automated diagnosis tools [41]. The authors concluded that the accuracy of the algorithm permits the use of the deep learning AI software in a wide range of applications in the future [41].

On the other hand, GE et al. analyzed a modality for digital histologic assessment of hepatic fat deposition mouse [42]. The study’s protocol described two phases: automated identification of digital images obtained from hepatic biopsy samples from mouse; automated diagnosis of the fraction of the identified area showing Oil Red O staining [42]. The automatization method was demonstrated to be rapid, with an average time per specimen of less than 3 min per, highly reproducible, and the area fraction was significantly associated with triglyceride deposition in the liver (*p* < 0.01) [42]. Further, Sethunath et al. analyzed the accuracy of machine learning modalities for diagnosing macro- and microsteatosis of fatty liver disease in murine models [43]. A fully automated diagnosis of steatosis was obtainable in murine liver biopsies images with outstanding accuracy [43].

### 3.3. NAFLD Diagnosis, Staging, and Risk Stratification Using Electronic Health Records

Electronic health records (EHR) are a digital systematized collection of patients’ electronically-stored health information hosted and shared through network-connected, enterprise-wide information systems [44]. We found seven articles analyzing the possibility of automated diagnosis, staging, and risk stratification of NAFLD based on EHR (Table 5).

Using an algorithm based on natural language processing (NLP) to analyze unstructured data Van Vleck et al. evaluated the possibility NLP for the identification of NAFLD patients with and to further analyze patterns of disease progression [45]. The protocol of the study included 38,575 individuals, registered in the NLP system, which were enlisted in the Mount Sinai BioMe cohort. The NLP system was used to find NAFLD imagistic diagnosis in radiology reports that was not reported in parallel in clinical notes, and physician opinion of progression of NAFLD to NASH or liver cirrhosis was also automatically analyzed [45]. The results demonstrated that NLP had better results than previous automated selection tools and breakdowns of essential information that could have slowed or prevented later disease progression were automatically found by the software [45].

Further, Corey et al. analyzed the possibility of an automated algorithm for NAFLD classification to be implemented in order to develop large-scale longitudinal cohorts [46]. The study reported that automated classification was better than other software’s using only the ICD-9 data with an AUC of 0.85 against 0.75 (*p* < 0.0001) [46]. The authors concluded that their method is simple to develop, easy to use, does not need individual training for the personal using the tool, and can be rapidly applied in order to create EHR cohorts of individuals with NAFLD [46].

In order to analyze and validate a machine learning method for an automated diagnosis of NAFLD, Yip et al. evaluated 922 individuals from a population-based screening study where the diagnosis of NAFLD was performed using proton-magnetic resonance spectroscopy [47]. The study protocol provided 23 routine clinical and laboratory parameters, including high-density lipoprotein cholesterol (HDL-C), triglyceride, alanine aminotransferase (ALT), hemoglobin A1c (HbA1c), white blood cell count, and presence of increased blood pressure [47]. NAFLD ridge score presented an area under the ROC curve of 0.87 (95% confidence interval, 0.83–0.90) and 0.88 (0.84–0.91) in the training and validation groups, and attained a sensitivity of 92% (86%–96%) and specificity of 90% (86%–93%) [47]. 

Perveen et al. evaluated a decision tree based method for NAFLD risk stratification using EHR [48]. The results confirmed that the algorithm is an accurate tool for physicians in their initiative for automated risk management of NAFLD patients using risk factors and specific clinical parameters, which are not direct indicators of NAFLD [48].

Katsiki et al. used machine learning techniques in a study on 31 NAFLD patients diagnosed on the histopathologic exam and 49 healthy controls in order to find an automated diagnosis alternative to biopsy, using the serum level of lipids, glycans, and other biochemical parameters [49]. Following the study protocol, the identification of 365 lipids, 61 glycans, and 23 fatty acids was performed using liquid chromatography mass spectrometry (LC-MS), where 10 lipid species had the power for a precise diagnosis of liver fibrosis with a 98% accuracy [49]. The conclusion was that the automated method of NAFLD and liver fibrosis diagnosis based on novel algorithmic models utilizing lipids, hormones, and glycans is a feasible alternative for liver biopsy or hepatic imaging [49].

A prediction model using machine learning algorithms was evaluated by Islam et al. on 994 patients with chronic liver disease [50]. The study protocol provided random forest (RF), SVM, artificial neural network (ANN), and logistic regression (LR) as prediction techniques for NAFLD [50]. The results demonstrated that the logistic regression method demonstrated the best accuracy of 76.3%, sensitivity of 74.1%, and specificity of 64.9% using EHR, compared with the other techniques that were used [50]. Further, Fialoke et al. used the EHC from Optum Analytics in order to evaluate an automated system prediction of NASH in NAFLD patients [51].

Alanine aminotransferase, aspartate aminotransferase, platelet counts, and type 2 diabetes status were analyzed by the automated software [51]. This study reported that cross-validated AUROC of various models ranged from 83–88%, making the method an accurate tool for clinicians and helping them in raising awareness on the upcoming evolution of NAFLD and further starting a tailored treatment scheme [51].

## 4. Discussion

Although the first studies about methods of an automated NAFLD diagnosis were published more than a decade ago, to our current knowledge, this is the first systematic review to evaluate the implementation of a complete automated NAFLD diagnosis, staging, and risk stratification using the available artificial intelligence-based software. Furthermore, in comparison with other reviews, we analyzed not only the imagistic methods but also the digital pathology methods and an EHC-based NAFLD diagnosis.

All the studies included in this systematic review show that their method is accurate, and it can be implemented in clinical practice and, also, in clinical trials. Although the excellent progress, the process of implementation in clinical practice is slow due to the lack of visibility of the studies and the skepticism of clinical personnel in the overall process of digitalization and automatization. Considering the unexpected effects of the 2020 COVID-19 crisis on the 3rd decade of the 21st century, which imply a comprehensive shortage of healthcare staff, including radiology, internal medicine, gastroenterology, general practitioners, and pathology specialists transferred in more urgent positions, a rapid implementation of an automated diagnosis software for chronic hepatic disorders is needed.

Because the expectation for a high precision medicine is rising, but at the same time, the reimbursements are dropping worldwide as a consequence of limited funds for healthcare providers, the transition from traditional pathology to digital pathology is a mandatory step forward. Our search identified sufficient data demonstrating that a digital pathology diagnosis and staging is now possible for NAFLD patients.

Furthermore, lockdown laws, social distancing, and the lack of personal protective equipment are forcing the healthcare staff and, also, the non-critical patients to avoid hospitals and to try to obtain an online telemedicine platform consultation.

The COVID-19 pandemic lockdowns are setting a new global artificial intelligence rush that accelerates all implementation attempts of an automated diagnosis in chronic, non-urgent disorders, including NAFLD. If initially, AI was reserved for simple operations, at present, the AI approach is expanding into areas that were previously thought to be exceedingly complicated for computer software and was thought to be managed by exclusively human experts.

Future testing and validation of the available automated NAFLD diagnosis methods depend on international collaboration and on a confident attitude of major healthcare corporations in adopting promising technologies.

Our systematic review has several limitations, which should be further discussed. The best method for NAFLD diagnosis is the histopathological examination, which is the current gold standard. Unfortunately, a considerable percent of the studies included in this review used common imagistic methods, which could overestimate the diagnosis of NAFLD. Another limitation of our systematic review is the insufficient studies about the EHR-based diagnosis of NAFLD, only seven studies. Our search included only studies about completely automated methods exclusively for NAFLD diagnosis, staging, and risk stratification; we excluded studies analyzing automated interpretation of abdominal imaging using ultrasound, CT, or MRI.

The metabolic-dysfunction-associated fatty liver disease (MAFLD) is a recently defined concept and was previously named NAFLD. MAFLD diagnosis criteria include hepatic steatosis, overweight/obesity, type 2 diabetes mellitus, or confirmed metabolic risk abnormalities. Unfortunately, current literature presents limited evidence about automated MAFLD diagnosis, and the vast majority of studies use the previous term.

## 5. Conclusions

We found significant evidence demonstrating that implementing a complete automated system for NAFLD diagnosis, staging, and risk stratification is currently possible, considering the accuracy, sensibility, and specificity of available AI-based tools. 

## Figures and Tables

**Table 1:** Studies assessing artificial intelligence-based imaging.

First Author	Publication Year	Country	Study Design	Total Patients	Mean Age (Years) (Mean ± Sd) /(Range)	Diagnosis	Sex (% Male)	Main Findings
Cao et al. [15]	2019	China	Cohort study	240	–	Nonalcoholic fatty liver disease (NAFLD)	54.58% male	For the diagnosis of NAFLD, envelope signal and grayscale values are very important. Deep learning presented the best results in assessing the severity of NAFLD.
Acharya et al. [16]	2016	Malaysia	Retrospective cohort study	150	22–79 years	Fatty liver disease	50%	A new technique which uses a probabilistic neural network (PNN) classifier, that can automatically identify NAFLD with an accuracy of 97.33%, specificity of 100.00%, and sensitivity of 96.00%.
Biswas et al. [17]	2017	Portugal	Cohort study	63	–	Normal (*n* = 27) Fatty liver disease (*n* = 36)	–	Deep Learning-based Symtosis™ system is reliable and stable in comparison with support vector machine (SVM) and extreme learning machine (ELM) for the ultrasound categorization of hepatic tissue and risk stratification of normal and abnormal hepatic images containing hyper- and hypoechoic areas.
Ribeiro et al. [18]	2014	Portugal	Cohort study	74	–	Normal (*n* = 38) Hepatic steatosis (*n* = 36)	–	A new computer-aided diagnosis (CAD) system reported values of 93.33% for accuracy, 94.59% for sensitivity, and 92.11% for specificity, utilizing the Bayes classifier for steatosis detection and classification.
Kuppili et al. [19]	2017	Portugal	Cohort study	63	–	Normal (*n* = 27) Fatty liver disease (*n* = 36)		A tissue characterization system based on a class of Symtosis for risk stratification of ultrasonographic hepatic images demonstrates superior performance compared to SVM.
Nagy et al. [20]	2015	Romania	Cohort study	228	44 ± 11.38	Mild hepatic steatosis (71.92%) Moderate-severe hepatic steatosis (28.08%)	51.32% male	The development of an image analysis software that depicts three local intensity parameters: the coefficient of variation of luminance (CVL), the median luminance (ml), and the hepato-splenic attenuation index (HSAI) from regions of interest (ROI) in the ultrasound image and analyses their depth variation. The proposed computer analysis method was found to be of use for initial non-invasive assessment and grading of hepatic steatosis, with the added advantage of reduced complexity and accessibility for the computations.
Subramanya et al. [21]	2014	India	Cohort study	53	–	Normal liver subjects (*n* = 12) Fatty liver disease (*n* = 41) ‑ mild (*n* = 14) ‑ moderate (*n* = 14) ‑ severe (*n* = 13)	–	A computer-aided diagnosis (CAD) system for assessing hepatic steatosis grading, mainly mild, moderate, and severe fatty liver, as well as normal liver tissue was developed according to the visual interpretations of radiologists, that selected region of interests (ROIs) from within the liver and from the diaphragm region, in each image. The features of selected ROIs pertaining texture were three-way combined in order to obtain ratio features, inverse ratio features, and additive features. A DEFS (differential evolution feature selection) algorithm and a SVM have been used. The computer-aided diagnosis system had the potential of being of use to the radiologists in order to asses steatosis grades.
Mihailescu et al. [14]	2013	Romania	Cohort study	120	–	Normal liver subjects (*n* = 10) Fatty liver disease (*n* = 110) ‑ mild (*n* = 70) ‑ moderate (*n* = 33) ‑ severe (*n* = 7)	–	Random forests (RF) was superior to support vector machine (SVM) classifiers and proved the capability of this classifier to perform well without feature selection. On the other hand, the performance of the SVM classifier was low without feature selection, which improved greatly after feature selection.
Han et al. [22]	2020	USA	Single-center prospective study	204	NAFLD (52 years ± 14) Controls (46 years ± 21)	Non-alcoholic Fatty liver disease (*n* = 140) Controls (*n* = 64)	Non-alcoholic Fatty liver disease (58.57% male) Controls (65.63%)	Liver radiofrequency ultrasound data contains bountiful information regarding livers’ microstructure and its composition. Deep learning can utilize these informationx in order to to assess for the presence of nonalcoholic fatty liver disease (NAFLD). Deep learning algorithms utilizing radiofrequency ultrasound data are accurate both for diagnosing NAFLD and also for the quantification of hepatic fat fraction, given that other causes of steatosis were excluded.
Graffy et al. [23]	2019	USA	Retrospective cohort study	9552	57.2 years ± 7.9	No liver fat (*n* = 5584) Mild steatosis (*n* = 4948) Moderate steatosis (*n* = 1025) Severe steatosis (*n* = 112)	44.36% male	The liver fat quantification tool, CT-based and fully automated, allows the population-based assessment of hepatic steatosis and NAFD, giving objective data that pairs well with data obtained by manual measurement. In this asymptomatic screening cohort, the prevalence of liver steatosis, of at least mild grade, was more than 50%.
Jirapatnakul et al. [24]	2019	USA	Cohort study	333	65 years (IQR 54–69 years) 61 years (IQR 57–66 years)	Liver disease subjects (*n* = 24) Normal liver function subjects (*n* = 319)	62.5% male 64.6% male	Automated measurements of liver attenuation in CT scans from patients with hepatic disease, can be utilized in identifying moderate to severe hepatic steatosis. The automated method outlines a set region within the liver, located below the right lung and utilizes statistical sampling techniques to elimitate all non-liver parenchyma.
Huo et al. [25]	2019	USA	Cohort study	246	–	NAFLD	–	An automatic ROI-based measurement (ALARM) method for estimating the liver attenuation was developed by combining DCNN and morphological operations, achieving an “excellent” accord with manual estimation of hepatic steatosis. The entire pipeline was implemented as a Docker container, enabling the software users to estimate the liver attenuation in five minutes per CT scan.

**Table 2:** Animal experimental studies Assessing Artificial Intelligence Based Imaging.

First Author	Publication Year	Country	Study Design	Total Patients	Mean Age (Years) (Mean ± Sd) /(Range)	Diagnosis	Sex (% Male)	Main Findings
De Rudder et al. [26]	2019	Belgium	Experimental study	20	–	NOD.B10 *foz/foz* and WT mice fed with high-fat diet C57B16/J mice fed with a fat-rich choline-deficient diet or fat-rich diet	100%	The automated procedure was able to assess and measure the grade of macrovesicular steatosis, mixed inflammation, and pericellular fibrosis in steatohepatitis induced by CDAA. The procedure represents a promising quantitative technique with high-throughput, in order to rapidly evaluate the NAFLD activity in large preclinical studies, and for accurate survey of disease evolution.
Starke et al. [27]	2010	Germany	Experimental study	151	–	German Holstein dairy cows–normal	0%	The CAUS methodology and software for digitally evaluating ultrasonographically obtained hepatic images could be accessible for noninvasive screening of hepatic steatosis in dairy herd health programs. Utilization of single parameter linear regression equation can possibly be optimal for practical applications.
Acorda et al. [28]	2011	Japan	Experimental study	158	–	Holstein-Friesian cows ‑ normal liver (*n* = 117) ‑ fatty infiltration of the liver (*n* = 41)		Fatty infiltration diagnosis was best performed using digital analysis, obtaining the highest sensitivity, specificity, accuracy, and PPV/NPV, followed by ultrasonography.

**Table 3:** Studies assessing automated digital pathology.

First Author	Publication Year	Country	Study Design	Total Patients	Mean Age (Years) (Mean ± Sd) /(Range)	Diagnosis	Sex (% Male)	Main Findings
Griffin et al. [29]	2017	United Kingdom	Review	**–**	**–**	**–**	–	Combining whole slide imaging with other digital tools such as digital dictation, specimen trackin, and barcoding, can simplify the histopathology workflow, from specimen accession to report sign-out, enhancing the safety, quality, and efficiency of histopathology departments.
Bera et al. [30]	2019	USA	Review (Opinion)	–	–	–	–	The opportunity of digitalization whole-slide tissue images has contributed to the appearance of artificial intelligence and machine learning tools in digital pathology, enabling the search for subvisual morphometric phenotypes, hopefully improving patient management.
Stathonikos et al. [31]	2020	The Nederlands	Review	–	–	–	–	The 2020 COVID-19 crisis led to several implications affecting healthcare providers, including staffing shortages and the necessity to work from home. An asset digital diagnostic could allow pathologists, residents, molecular biologists, and pathology assistants to participate in the diagnostic process.
Ortega et al. [32]	2020	USA	Review	–	–	–	–	Using hyperspectral imaging (HSI) and multispectral imaging (MSI) technologies can add spatial information for creating computer-aided diagnostic tools for histological samples, both stained and unstained. HIS/MSI is associated with new possibilities in histological samples evaluation such as digital staining or mitigating the variability of digitized samples between different laboratories, compared to the traditional RGB analysis method.
Mendelsohn et al. [33]	2016	USA	Review	–	–	–	–	CYDAC (cytophotometric data converter) is a new modality, depending on computer analysis of cell images that can be applied to blood cell and chromosome discrimination.
								This specific modality to pictorial data processing can possibly have utilizations in other scientific areas.
Forlano et al. [34]	2020	United Kingdom	Retrospective cohort study	246	51 (19–77)	Biopsy-proven NAFLD	69%	An algorithm was developed through machine learning, by utilizing liver specimens obtained through biopsy from NAFLD patients, for quantifying the ammount of fat, inflammation, ballooning, and collagen. The algorithm proved predictability and sensitivity for the detection of modifications compared with traditional scores, in a cohort of paired specimens from liver biopsies.
Munsterman et al. [35]	2019	The Nederlands	Case-control study	79	–	Control subjects (*n* = 18) NAFLD subjects (*n* = 61)	–	Validation study for the development of a digital automated system for quantification of steatosis on whole-slide images (WSIs) of liver tissue. This algorithm can also be applied when appraising the degree of liver steatosis is warranted, such as clinical trials assessing the effectiveness of new therapeutic interventions in NAFLD.
Segovia-Miranda et al. [36]	2019	Germany	Cohort study	25	68 (54–85)	Normal control (*n* = 6)	33%	Computational simulations multiphoton imaging, and three-dimensional digital reconstructions were applied, achieving geometrical and functional spatially rendered models of human liver tissue from various non-alcoholic fatty liver disease (NAFLD) stages. The proposed models can define quantitative multiparametric signatures for cells and tissues, assessing disease progression and providing new patophysiologic insights into NAFLD.
					36.5 (29–68)	Healthy obese (*n* = 4)	25%	
					42 (34–51)	Steatosis (*n* = 8)	63%	
					51 (39–58)	Early NASH (*n* = 7)	14%	
Vanderbeck et al. [37]	2015	USA	Case-control study	47	–	Normal liver histology subjects (*n* = 20) NAFLD subjects (*n* = 27) of varying severity: ‑ simple steatosis (*n* = 19) ‑ NASH (*n* = 8)	–	Automatic quantification of cardinal NAFLD histologic lesions can be accessible and may possibly be used for further developing other automated methods for pathologists, in order to assess NAFLD biopsies in clinical practice and clinical trials.
Teramoto et al. [38]	2020	Japan	Cohort study	80	–	NAFLD subjects (*n* = 79)	–	A method linking topological data analysis with linear machine learning techniques was devised and tested for classifying liver tissue images, using Matteoni classification, into NAFLD subtypes.
Gawrieh et al. [39]	2020	USA	Cohort study	18	–	NAFLD (liver biopsies)	–	An integrated artificial intelligence-based automated tool was developed and tested to identify and evaluate hepatic fibrosis and its patterns in liver biopsies of NAFLD.
Vanderbeck et al. [40]	2014	USA	Case-control study	47	–	Normal liver histology (*n* = 20) NAFLD (*n* = 27)	–	An automatic classification of steatosis was proposed that included the basic features of NAFLD compared to other regions that appear as white in images of liver biopsies samples stained with hematoxylin and eosin (macrosteatosis, portal arteries, portal veins, central veins, sinusoids, and bile ducts). The precise identification of microscopic anatomical landmarks in liver samples, can ease critical ensuing tasks, (locating other histological anomalies)

**Table 4:** Animal experimental studies assessing artificial intelligence-based imaging.

First Author	Publication Year	Country	Study Design	Total Patients	Mean Age (Years) (Mean ± Sd) /(Range)	Diagnosis	Sex (% Male)	Main Findings
Ramot et al. [41]	2020	Israel	Experimental study	32 mice	–	Experiment with High-fat diet (HFD): 16 C57BL/6J male mice, divided in 6 groups: normal diet; HFD 10% (wt/wt) dietary broccoli; HFD10% (wt/wt) dietary broccoli stalks. Experiment with High cholesterol and cholate diet (HCD): 16 C57BL/6J male mice, divided in 4 groups: normal diet; diet high in fat high cholesterol (1%) and cholate (0.5%) (HCD; atherogenic diet); HCD 15% (wt/wt) dietary broccoli; HCD 15% (wt/wt) dietary broccoli stalks.	100%	A deep learning AI algorithm was developed by utilizing glass slides of liver from mice models for nonalcoholic fatty liver disease, quantifying hepatic fatty vacuoles, while differentiating them from lumina of liver blood vessels and bile ducts. Utilizing deep learning algorithms for difficult assessments utilized in microscope-based pathology can advance outputs of workflows regarding toxicologic pathology.
Ge et al. [42]	2010	USA	Experimental study	42 mice		Male C57BL/6J, ob/ob and db/db mice divided into 7 groups: ‑ Control ‑ 10% Ethanol ‑ 14% Ethanol ‑ 18% Ethanol ‑ high-fat diet (HFD) ‑ Ob/ob ‑ Db/db	–	A rapid and reproducible modality for histologic assessment of hepatic fat deposition in different models of hepatic steatosis in mice, based on quantitative digital analysis of Oil Red O (ORO) accumulation in fresh-frozen hepatic sections was developed. The process involved defining appropiate regions for analysis, followed by digital photographic imaging of these regions and subsequently by the digital determination of the portion of the identified area (Area Fraction) exhibiting ORO staining.
Sethunath et al. [43]	2018	USA	Experimental study	27 mice	–	Normal liver histology (*n* = 9) Mild steatosis (*n* = 10) Moderate steatosis (*n* = 4) Severe fatty liver (*n* = 4)	–	Accurate identification of macro- and microsteatosis in FLD in mice is critical in understanding the pathophysiology of the disease, and in the detection of potential hepatotoxic signatures which can be applied in drug development and quantifying the effects of different therapies. Automated classifiers were developed employing both image processing techniques and machine learning techniques, in order to study the correlation between automated quantification of macrosteatosis and semi-quantitative grades given by expert pathologists. The developed classifier proved high accuracy and sensitivity for indentifying macrosteatosis in fatty liver disease in mice.

**Table 5:** Studies assessing artificial intelligence-based technologies on electronic health records.

First Author	Publication Year	Country	Study Design	Total Patients	Mean Age (Years) (Mean ± Sd) /(Range)	Diagnosis	Sex (% Male)	Main Findings
Van Vleck et al. [45]	2019	USA	Retrospective cohort study	12,934	59.8	NAFLD subjects (*n* = 2281)	42%	The use of NLP (natural language processing) algorithms to appropiately evaluate unstructured data in EHR has been extensively documented. NLP-based approaches were more detailed in defining NAFLD within the EHR compared to ICD/text search-based methods. NLP algorithms promoted better analysis of knowledge flow between physician and allowed identifying certain breakdowns that conducted to key information misplacement, information that could have been used in slowing disease progression.
					59.5	Control subjects (*n* = 10653)	39%	
Corey et al. [46]	2016	USA	Retrospective cohort study	620	61.6 (12.7)	NAFLD subjects (*n* = 444)	44%	The NAFLD classification algorithm designed within the electronic medical record (EMR) for establisment of longitudinal large-scale cohorts was better than ICD-9 billing data by itself. This method proved simple to establish and use through various institutions.
					58.7 (15.2)	Not NAFLD subjects (*n* = 176)	46%	
Yip et al. [47]	2017	Hong Kong	Retrospective cohort study	922	50.7 ± 9.5	NAFLD subjects (*n* = 264)	54.2%	Six factors (ALT, HDL-C, triglyceride, HbA1c, white blood cell count, and hypertension) were included in a machine learning model based on laboratory parameter values, in order to identify NAFLD in the general population. The NAFLD ridge score was demonstrated to be a simple modality that can be compared to current NAFLD scores currently used in epidemiological studies to exclude patients with NAFLD
					47.0 ± 10.8	Healthy subjects (*n* = 658)	37.4%	
Perveen et al. [48]	2018	Canada	Retrospective cohort study	40,637	61.2 ± 14.2	NAFLD subjects	40%	A decision tree-based method was proposed for risk-assessment related to NAFLD development and progression in the Canadian population. The method used electronic medical eecords by analyzing the presence of risk factors for NAFLD. Using the proposed application linked to the everyday medical checkup is likely to aid physicians in performing more informed decisions about their patients’ management with NAFLD, while reducing healthcare spending.
Katsiki et al. [49]	2019	Greece, Italy, and United Kingdom	Editorial	–	–	–	–	Using lipidomic and metabolomic methods can contribute as diagnostic biomarkers because NAFLD is a metabolic disease. A pilot case–control study reported the benefits of utilizing non-invasive methods based on omics and supervised machine learning for diagnosing and treating NAFLD. These models may serve as a useful, non-invasive model, representing an attractive alternative to liver biopsy.
Islam et al. [50]	2018	Taiwan	Case-control study	994	62.1 ± 12.55	Fatty liver subjects (*n* = 593)	46.37%	The study reports that machine learning models, especially logistic regression model are associated with an improved accuracy in predicting FLD, based on data found in electronic medical records and could be able to be an essential tool for clinical decision making.
					62.07 ± 13.52	Non-fatty liver subjects (*n* = 401)		
Fialoke et al. [51]	2018	USA	Retrospective cohort study	108.139	57.60 (13.43)	NASH subjects (*n* = 17,359)	41.22	Longitudinal statistical properties of lab-based parameters (e.g., mean of all ALT values) were used to create supervised a machine learning model trained on NASH and healthy patients. The proposed model performed better than most of the non-invasive techniques currently in practice for diagnosing NASH.
					61.36 (18.34)	Healthy subjects (*n* = 17,590)	40.17	
					57.30 (14.65)	Subjects at risk for NAFL (*n* = 73,190)	39.65	
